# Geochemical Characteristics of Rare-Metal, Rare-Dispersed, and Rare-Earth Elements and Depositional Environments in the Shanxi Formation Coal, Huainan Coalfield, Anhui, China

**DOI:** 10.3390/ijerph20031887

**Published:** 2023-01-19

**Authors:** Weicheng Wang, Liugen Zheng, Zhiwei Wu, Qian Zhang, Xing Chen, Yongchun Chen, Liqun Zhang

**Affiliations:** 1 School of Resource and Environment Engineering, Anhui University, Hefei 230601, China; 2 National Engineering Laboratory of the Coal Mine Ecological Environment Protection, Huainan 232001, China

**Keywords:** Huainan coalfield, TREs, geochemistry, depositional environment

## Abstract

Coal, being one of the major energy sources for power generation, contains several critical trace elements. There is a growing scarcity and expense of these critical elements as a result of the increased demand and limitation of mining sources. To explore the geochemical characteristics of the rare-metal, rare-dispersed (scattered), and rare-earth elements (TREs) in coal, 25 coal seam samples of the Shanxi Formation in the Huainan coalfield were collected. The major element oxides, minerals, and TREs were analyzed by X-ray fluorescence spectroscopy (XRF), X-ray diffraction (XRD), and inductively coupled plasma-mass spectrometry (ICP-MS). The results revealed that the coal of the Shanxi Formation had ultra-low moisture and low ash yield and was medium–high-volatility with low sulfur content and high calorific value. Concerning minerals, the coal was mainly composed of kaolinite, illite, quartz, calcite, dolomite, and pyrite. Compared with Chinese coal and world hard coal, rare-metal element Li and rare-dispersed element Se were enriched, whereas Ga and Ta were only slightly enriched. The average content of REYs was 51.34 μg/g, which is lower than the average content of REYs in Chinese coal. It has the enrichment characteristics of light REYs. In the vertical direction, the content of most TREs was higher in the roof and floor of the coal seam and the parting, indicating that the sedimentary microenvironment plays an important role in controlling the migration and enrichment of elements. The experimental results of sequential chemical extraction and correlation analysis showed that the TREs in the Shanxi Formation coal mainly exist in a residual and carbonate bound state, and occur in clay minerals and carbonate minerals. The enrichment of Se may be due to its high organic form ratio. The C-value, B content, *w*(Sr)/*w*(Ba), and REY geochemical parameters indicated that the Shanxi Formation Coal seam was developed in a transitional, semi-saline, deltaic sedimentary environment. With their development affected by seawater, REYs in coal are greatly supplied by terrigenous clastics. The complex sedimentary environment is an important reason for the varying occurrence states of TREs in the Shanxi Formation coals.

## 1. Introduction

As a special sedimentary organic rock, the composition of coal is complex. Currently, more than 80 kinds of trace elements have been found in coal [1]. In addition to supplying 37% of the world’s electricity needs as the main energy source for power generation [2], coal is also regarded as the mining source of strategically important elements (Li, Ga, Ge, REE, etc.) [3,4,5]. These critical elements are becoming scarce and expensive due to the growing demand and the limitation of mining sources. The recovery of key elements from coal and its combustion by-products have attracted the attention of many researchers [6,7,8].

The “three types of rare mineral resources elements” (TREs) proposed by the China Geological Survey include rare-metal elements (Li, Be, Nb, Ta, Zr, Sr, Hf, Rb, and Cs), rare-dispersed (scattered) elements (Ga, Ge, In, Cd, Tl, Re, Se, and Te), and rare-earth elements (REY: 15 lanthanide elements containing Y and Sc) [9]. In recent years, coal deposits have attracted much attention as a potentially promising alternative raw material source for rare-metal, rare-dispersed, and rare-earth elements. Many scholars have discussed coal-measure mineral resources, including the types, abundances, development, and utilization grades of coal-measure metal minerals, the mechanism and law of enrichment and integration, and the occurrence states of metal elements [10,11,12,13,14,15,16]. The development and utilization of coal-based metallic minerals show great prospects. The European Commission’s recent list of critical metal resources in September 2020 mainly involves REEs, Ga, Sr, Nb, Hf, Ta, and 30 other kinds of raw materials with great economic and strategic value [17]. Rare-metal elements Li and Sr have been included in the list for the first time. Coal geologists have successively discovered coal mines that are abnormally enriched in associated metallic elements, for instance, the Shengli Coalfield, which is the largest Ge-coal deposit in the world; an anomalous enrichment of Ga in coal and a few potentially economic Ga-enriched coal deposits were discovered in the Jungar Coalfield, Inner Mongolia [18,19]. The extraction of rare-earth elements (REYs) from coal or fly ash has been of increasing interest in recent years due to their stable chemical properties. In addition, REYs are widely used as geochemical indicators of the syngenetic, diagenetic, and epigenetic processes affecting coal deposits and explaining the origin of coal-forming materials because of their consistent behavior and predictable fractionation patterns in different geochemical processes [20,21].

The study of rare-metal, rare-dispersed, and rare-earth elements in coal not only provides important coal geochemical, and coal mineralogical evidence for studying the geological genesis and regional geological, and historical evolution of coal seams but also represents a significant change in the way of coal utilization. However, based on the resource characteristics of coal-based metallic minerals, the related research also faces many difficulties and challenges. The paragenesis and occurrence of TRE in coals, as well as the reasonable development and utilization of such coals in the Huainan coalfield, have not been investigated in detail. In this study, new data and evidence of the characteristics of coal quality and the geochemical characteristics (abundance, enrichment, distribution, genesis, and modes of the occurrence) of rare-metal elements (Li, Be, Rb, Sr, Zr, Nb, Cs, Hf, and Ta), rare-dispersed elements (Ga, Ge, Se, Cd, and Ta), and rare-earth elements (REE and Y) were obtained on Late Permian bituminous coals from the Huainan Coalfield, Anhui, China. Our study also discusses the relationships between trace element abundances and depositional environments of coals. The results of the study can provide theoretical support for the rational development and utilization of the deep Shanxi Formation coal in Huainan coalfield.

## 2. Geological Setting

**Figure 1:**
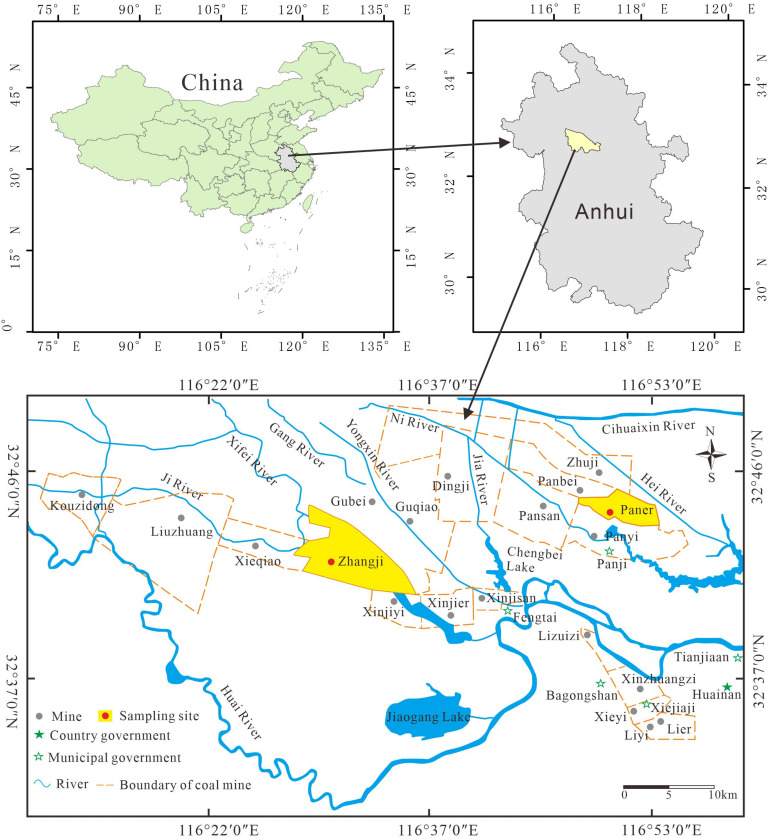
Location map of the Huainan Coalfield, China.

The Huainan coalfield is an important coal production area in east China, producing more than 100 million tons of coal per year. It is located in the northern Anhui province at the southeast corner of the North China Plate (Figure 1). The coalfield covers a total area of 3200 km^2^ and is 180 km in mean length and 15–25 km in mean width. It is a completely hidden area covered by loose layers in the Cenozoic. Since the Late Paleozoic, the Huainan coalfield has undergone a recurrence of marine transgression and regression, and has been under the combined effects of the Qinling-Dabie Orogenic Belt and the Tanlu strike-slip fault, leading to the formation of a complex and complete deltaic system developed on the offshore bay with unique structural evolutionary characteristics. The Carboniferous-Permian period was an important peat-forming period in the Huainan coalfield. The coal-bearing sequences in the Huainan Coalfield include the Benxi Formation of the Late Carboniferous; the Taiyuan Formation, the Shanxi Formation, the Lower Shihezi Formation, and the Upper Shihezi Formation of the Early Permian. The Shanxi, Lower Shihezi, and Upper Shihezi formations constitute the main mineable coal-bearing sequences in the Huainan coalfield [22,23]. With the development of mining, the Huainan Coalfield has now entered the mining stage of the deep Shanxi Formation coal seam.

As the late main mining seam of the Huainan coalfield, the Shanxi Formation is connected downward to the Taiyuan Formation and upward to the Lower Shihezi Formation. The Shanxi Formation began to deposit in the delta front, forming a set of lithologic assemblages of sandstone, shale, and coal [24]. Its main coal-bearing strata are coal seam 1 and coal seam 3. The Zhangji coal mine is located 20 km west of Fengtai County, Huainan City, at the southeast dip end of the Chenqiao backslope, which is a fan-shaped monoclinic structure with irregularly curved strata, covering an area of ~71 km^2^; the entire mining area is triangular. Coal seam 1 is the coal-bearing stratum of the Shanxi Formation, with a thickness of 0–8.28 m (average 6.52 m). The Paner coal mine is located in the Nihe Town, Panji District, Huainan City, Anhui Province. It is located at the end of the eastern uplift of the Panji Backslope of the Huainan Complex Oblique, with large- to medium-sized internal faults and folds and their development; the central part of the coal seam is oriented east–west. The overall shape is that of a fan, and the wellfield area spans ~35.14 km^2^. Coal seam 3 is the coal-bearing stratum of the Shanxi Formation, with a thickness of 1.28–9.17 m (average 5.07 m).

## 3. Sample Collection and Analytical Methods

**Figure 2:**
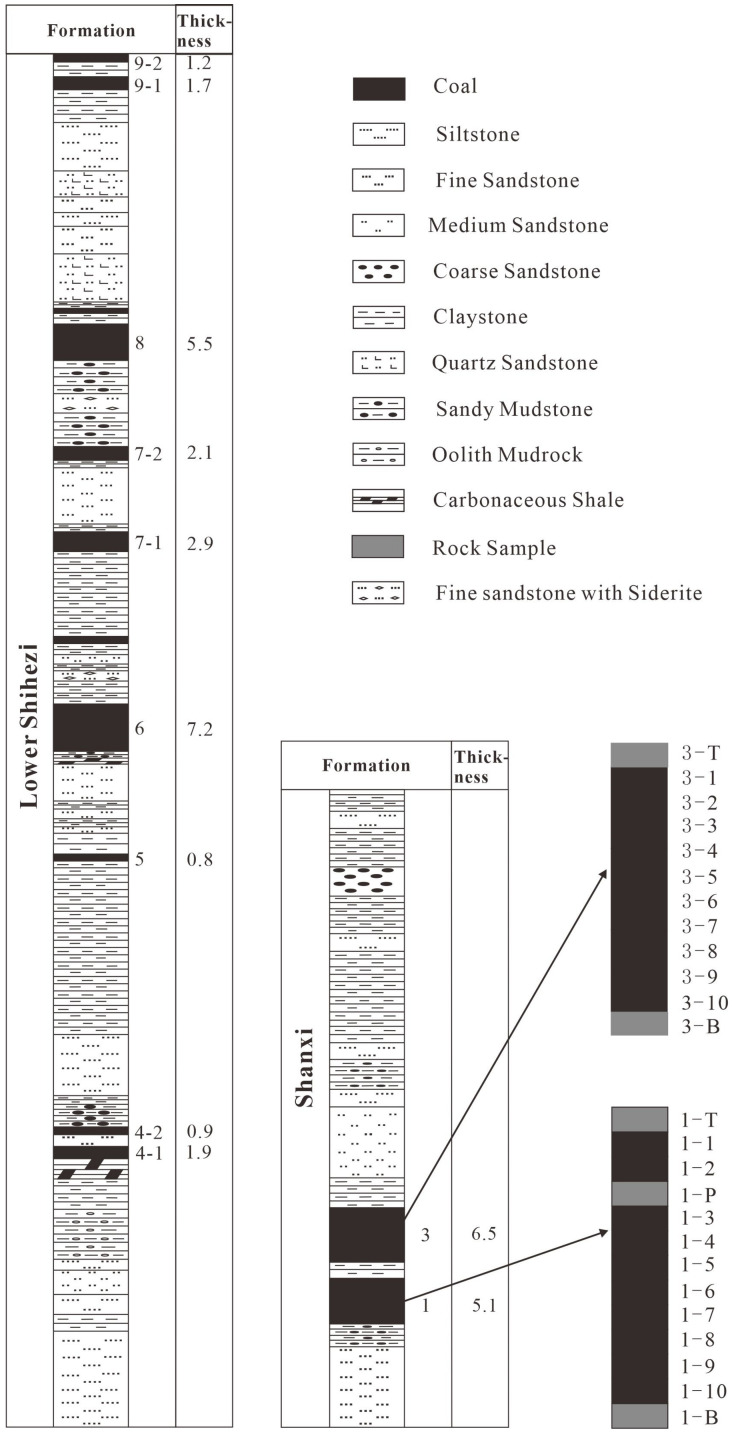
Generalized stratigraphic column and specific sampling points in the study area.

A total of 25 samples (including coal, roof, floor, and parting samples) were collected from the Shanxi Formation using a channel-profile sampling method following the Chinese National Standard Method (GB/T 482-2008) (Figure 2). Each coal bench sample was cut to dimensions of 10 cm wide, 10 cm deep, and 10 cm thick. The sampling interval from coal seam 1 of the Shanxi formation was 0.5 m. A total of 10 coal samples were collected from top to bottom numbered 1-1–1-10. One roof, one floor, and one parting samples were numbered 1-T, 1-B, and 1-P, respectively. The sampling points from coal seam 3 of the Shanxi formation were spaced 0.4 m apart, and 10 coal samples were collected, numbered 3-1–3-10 from the top to bottom. One roof sample and one floor sample were numbered 3-T and 3-B, respectively. To avoid sample contamination, oxidation, and loss of moisture, the collected samples were immediately sealed and stored in polyethylene bags. The coal samples were taken back to the laboratory, ground, passed through a 200-mesh sieve, and stored in labeled brown wide-mouth bottles.

According to the industrial analysis method of coal (GB/T 212-2008), the moisture (M), ash yield (A_d_), volatile matter (V), and calorific value (Q) of coal were measured by a fully automatic industrial analyzer (SDT-GA5000a, Sundy, Changsha, China). The content of total sulfur (S_t_) was determined by a SDS601 sulfur analyzer, following GB/T 214-2007, and the contents of sulfate sulfur (S_s_), pyritic sulfur (S_p_), and organic sulfur (S_o_) were determined following GB/T 215-2003.

Each sample was ashed at a high temperature of 815 °C, and loss on ignition was calculated. After tableting, the major oxides were determined by X-ray fluorescence spectrometry (XRF) (ZSX Primus II type, Rigaku Industrial Corporation, Tokyo, Japan). The phase mineral composition of the prepared samples was determined by XRD using the Smart-Lab 9 kW (Rigaku Industrial Corporation, Osaka, Japan), with acceleration voltage ≤ 45 kV, tube flow ≤ 200 mA, and power ≤ 9 kW; each XRD pattern was recorded over a 2θ interval of 5–75°, with a step size of 0.02°. The 2θ angle indication error was 0.017°, the resolution was 27%, and the diffraction intensity stability was 1.1%.

The elements of TREs, B, and Ba in the coal were determined by ICP-MS (Agilent 7500cx, Agilent, Palo Alto, CA, USA). The operating parameters of the suppressor were: RF power 1500 W, auxiliary gas (Ar) flow 0.90 L/min, and atomizer (Ar) flow 0.25 L/min, and the error analysis was −1.775 ± 2.745. The chemical forms of TREs were analyzed by a sequential chemical extraction procedure [25] (Table 1); three parallel samples were chosen, with a recovery level of 97.2–101.7%.

## 4. Results and Discussion

### 4.1. Physicochemical Characteristics of Coal

The moisture (M) content of the coal in the Shanxi formation was 1.20–2.93%, with an average content of 1.78%; the ash yield (A_d_) content range was 3.45–29.04%, and the average content was 10.89%. The volatile matter (V_daf_) content range was 28.65–38.44%, and the average content was 32.76%; the total sulfur content range was 0.32–0.71%, and the average content was 0.50%. Pyritic sulfur and organic sulfur were the main forms of sulfur in the Shanxi Formation coal, while sulfate sulfur was the least common form. The average of the Shanxi formation coal calorific value was 27.59 MJ/kg (Table 2). According to Chinese National Standards MT/850-2000, MT/T 849-2000, and GB/T 1522.4-2010, the coal from the Shanxi Formation in the deep region of Huainan could be classified as ultra-low-moisture, low-ash-yield, medium–high-volatility, low-sulfur, and high-calorific-value coal.

The average of major oxides of coal in the Shanxi formation was in the order of SiO_2_ (5.43%) > Al_2_O_3_ (4.93%) > Fe_2_O_3_ (1.24%) > CaO (0.83%) > MgO (0.46%) > TiO_2_ (0.27%) > K_2_O (0.25%) > Na_2_O (0.07%) > P_2_O_5_ (0.02%). The percentages of major-element oxides compared with the average values for Chinese coals are presented in Table 3. An average of major-element oxides was lower than that of average Chinese coals, mainly due to their low ash yield. The ash yield belonged to SiO_2_-Al_2_O_3_-Fe_2_O_3_-CaO, indicating that more detritus minerals were transported to the study area and deposited on the coastal delta plain where it was open to clastic influx. SiO_2_ and Al_2_O_3_ were the main element oxides in the Shanxi Formation coal, indicating that the minerals in the raw coal were composed of clay minerals (such as kaolinite and illite) and quartz. The high Fe_2_O_3_ content indicated that the minerals in the coal may be composed of sulfide minerals (e.g., pyrite) and carbonate minerals (e.g., rhodochrosite). The depositional environment of the peat accumulation phase in coal can be indicated by the [w(CaO) + w(MgO) + w(Fe_2_O_3_)]/[w(SiO_2_) + w(Al_2_O_3_)] ratio (C) [26,27] when 0.03 < C < 0.22, indicating that the coal seam was formed in a terrestrial marsh environment uninfluenced by seawater. However, 0.22 < C < 1.23 could imply transition areas from terrestrial to shallow marine, with coal seam development having been affected by seawater [28]. The C in the coal of the Shanxi formation was 0.14 and 0.35 with an average of 0.25, indicating that the Shanxi formation coal formed in a seawater transgressive environment, where a marine influence into paleomires could be common.

**Figure 3:**
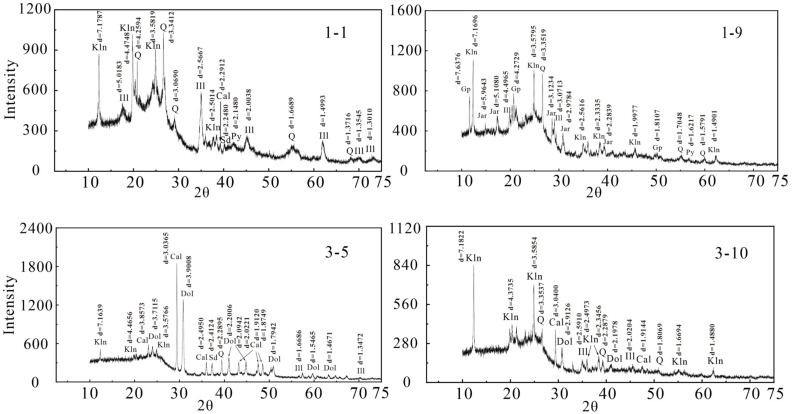
X-ray diffraction pattern of coal samples from Shanxi Formation in the Huainan coalfield. Kln—kaolinite; Q—quartz; Ill—illite; Cal—calcite; Dol—Dolomite; Sd—Siderite; Py—pyrite; Gp—Gypsum; Jar—Jarosite.

The results of X-ray diffraction (XRD) (Figure 3) revealed that the main minerals in the coal of the Shanxi Formation in the deep part of Huainan were clay minerals (kaolinite, illite) and oxide minerals quartz, both of which were mainly derived from the weathering of quartzite and sandstone in the Funiu and Dabie ancient land provenance [29]. The carbonate minerals were mainly calcite, dolomite, and a small amount of siderite; pyrite was the only sulfide mineral found in coal. Pyrite generally reflects the reductive sedimentary environments and often occurs in coal seams related to marine sedimentation [30]; in addition, there was a small amount of sulfate mineral gypsum and jarosite. The XRD results were consistent with the mineral types in the coal predicted by the ash composition.

### 4.2. Geochemical Characteristics of TREs

#### 4.2.1. Content Characteristics of TREs

The total quantity of rare-earth elements (REYs) in the coal of the Shanxi Formation in the deep part of Huainan coalfield ranged from 31.06 to 80.57 μg/g (on average, 51.34 μg/g), lower than the world hard coal average of 68 μg/g, and significantly lower than the average concentration of rare-earth elements in Chinese coal of 136 μg/g (Table 4). In general, the REY content of the Shanxi Formation in the deep part of Huainan coalfield was low and relatively unenriched. REYs were further divided into light, medium, and heavy fractions: LREY (La, Ce, Pr, Nd, and Sm), MREY (Eu, Gd, Tb, Dy, and Y), and HREY (Ho, Er, Tm, Yb, and Lu), respectively [31]. The average LREY, MREY, and HREY content of the Shanxi Formation coal in the deep part of Huainan was 42.24 μg/g (range: 25.19–71.73 μg/g), 7.68 μg/g (range: 2.43–16.08 μg/g), and 1.42 μg/g (range: 0.56–3.71 μg/g), respectively. The average value of LREY/MREY was 6.14 (3.10–11.52), revealing that LREYs were enriched relative to MREYs; the average value of MREY/HREY was 5.69 (2.94–10.11), revealing that MREYs were enriched relative to HREYs; the average value of LREY/HREY was 34.19 (18.47–68.08), revealing that LREYs were significantly more enriched relative to HREYs. The higher LREY/HREY and MREY/HREY may be because HREYs are more easily dissolved or leached by transgressive seawater or groundwater than LREYs [9].

**Figure 4:**
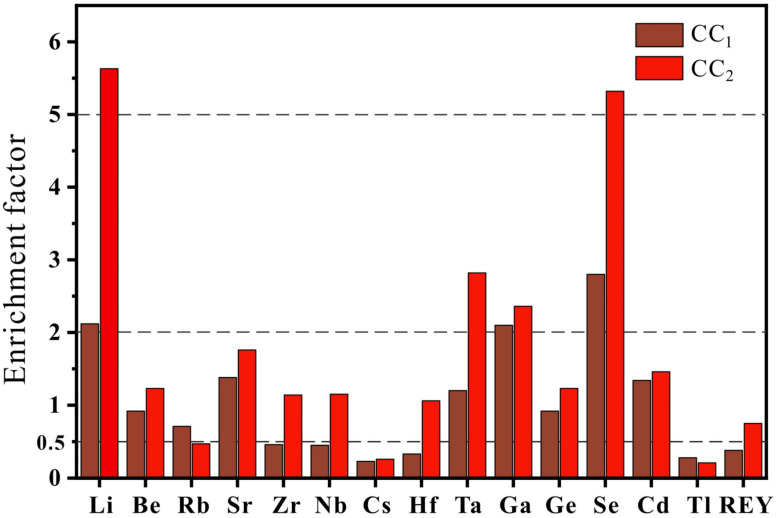
Ratio of the Shanxi Formation coal to Chinese coal and world coal in the Huainan coalfield. CC_1_—concentration coefficient = AM of this study/AM of Chinese coal. CC_2_—concentration coefficient = AM of this study/AM of world hard coal.

The test results of rare-metal and -dispersed elements (Table 4) were compared with the average content of elements in North China coal [32]. The average content of Li (1.54×), Rb (4.16×), Se (3.44×), and Ge (2.92×) was higher than that of the North China coal, while the average concentrations of Zr (0.22×), Nb (0.62×), Cs (0.67×), Hf (0.25×), and Tl (0.61×) were significantly lower than those of North China coal, and the average concentrations of the remaining elements were close to those of North China coal. The average concentration of rare-metal and -dispersed elements in the Shanxi formation coal is presented in Table 2 and are compared with the average of Chinese coal [33] and world hard coal [34]. The concentrations of elements in coal are characterized by the enrichment coefficient (CC= content of trace elements in coal/averages of trace elements in regional coal) [35]. According to the element enrichment coefficient resulting from comparison with the average value of Chinese coal (CC_1_) (Figure 4), Zr, Nb, Cs, Hf, Tl, and REYs in the Shanxi Formation coal were depleted (CC_1_ < 0.5). The enrichment coefficients of elements Be, Rb, Sr, Ta, Ge, and Cd were close to the average value of Chinese coal (0.5 ≤ CC_1_ ≤ 2), and Li, Ga, and Se were slightly enriched relative to Chinese coal (2 ≤ CC_1_ ≤ 5). Compared to world hard coal, the elements that showed loss (CC_2_ < 0.5) were Rb, Cs, and Tl. The enrichment coefficient of Be, Sr, Zr, Nb, Hf, Ge, Cd, and REYs was 0.5 ≤ CC_2_ ≤ 2, which was close to the world hard coal average. While Ta and Ga were slightly enriched (2 < CC_2_ ≤ 5), Li and Se were 5.63 and 5.32 times the world hard coal average, respectively, and were enriched in the Shanxi Group coal. In general, Li (67.56 μg/g) and Se (6.92 μg/g) were enriched, and Ga (13.67 μg/g) and Ta (0.79 μg/g) were slightly enriched in the coal of the Shanxi Formation in the Huainan coalfield.

#### 4.2.2. Vertical Distribution of TREs

**Figure 5:**
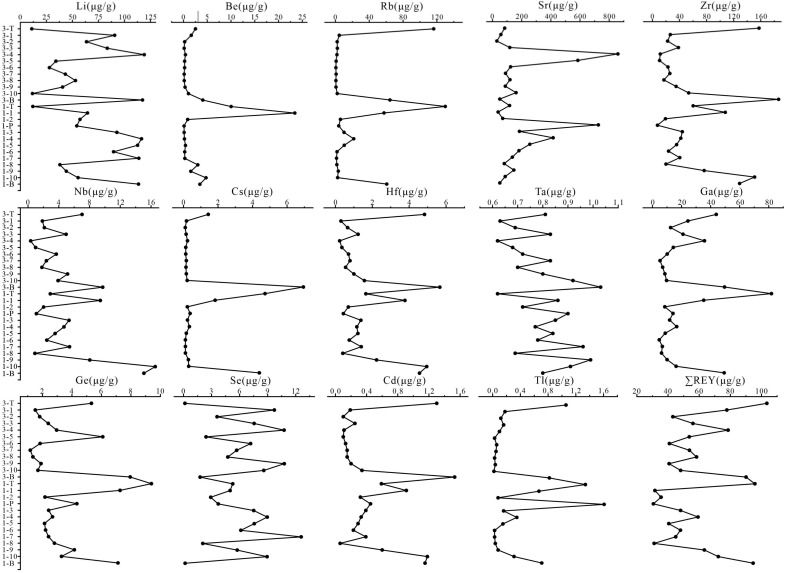
Vertical distribution of TREs in the deeply buried group Shanxi coal seam of Huainan coalfield (μg/g).

The spatial distribution characteristics of TREs in the Shanxi formation coal are discussed and presented in Figure 5. The rare-metal elements Be–Rb–Sr–Zr–Nb–Cs–Hf–Ta in the Shanxi formation coal showed a vertical distribution similar to the rare-dispersed elements Ga–Ge–Cd–Tl from coal seam 3 to coal seam 1. It is evident that the content of these elements in the roof, floor, and the parting of the coal seam was high. For example, the content of Zr in the roof and bottom of coal seam 3 was 157.40 μg/g and 186.40 μg/g; the content of Sr in gangue was 825.70 μg/g, higher than the average content of 193.40 μg/g in the coal. Previous studies also found more trace elements enriched at the top and bottom of the coal in the Huainan coalfield [36]. The enrichment of trace elements in the coal seam divide, top, and bottom slabs may be related to their inorganic affinity [37]. It may also be attributed to the leaching and transport during the rise or fall of the groundwater level and its flow [38]. In addition, the contents of rare-metal elements Li, Ta, and the rare-dispersed element Se varied greatly in the vertical section, showing a multi-peak change in depth. The content distribution in the same coal seam was also extremely uneven, showing a zigzag distribution. The content of REYs in the roof and floor of the Shanxi formation was higher than that in coal, but the total quantity of rare-earth elements in the parting was lower than that in coal. In addition, the content of REYs in the Shanxi Formation coal increased gradually from coal seam 1 to coal seam 3. Seawater intrusion into peat swamp affects the content and distribution of trace elements [39]. Furthermore, even in similar depositional environments, there are differences in the content of rare-earth elements of coal seams formed by different microenvironments [40]. The deep Shanxi formation coal in the Huainan coalfield was developed in a transitional depositional environment from the submerged deltaic plain to the lower deltaic plain, where the influence of seawater gradually weakened and the increasing influence of input from land-derived debris was the crucial reason for the differences in the content of TREs in the vertical direction.

### 4.3. Occurrence State of TREs

The occurrence state of elements in coal is very complicated and is affected by the depositional environment, among other factors. Modes of occurrence of an element indicate how this element is chemically bound or physically distributed throughout the coal [36,41,42]. The Pearson correlation coefficient of the TREs with ash, total sulfur content, major elements, and selected elements or element combinations is presented in Table 5, and is combined with the test results of a sequential chemical extraction experiment to explore the occurrence of TREs in the Shanxi formation coal.

#### 4.3.1. Rare-Metal Elements

**Figure 6:**
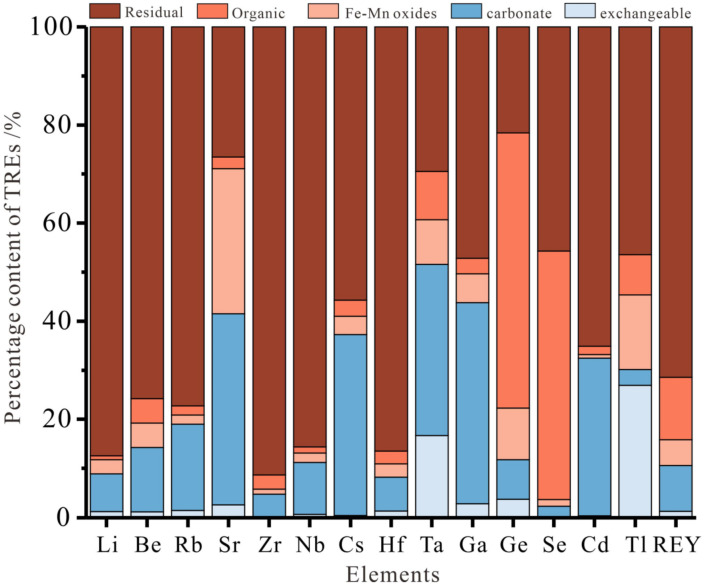
Experimental results of sequential chemical extraction of TREs.

According to the result of the sequential chemical extraction experiment (Figure 6), the residual mass fractions of Zr, Nb, and Hf were 91%, 86%, and 86%, respectively. Zr, Nb, and Hf were positively correlated with ash (r = 0.726, 0.623, and 0.710) and Al (r = 0.613, 0.596, and 0.562). The correlation coefficient between Ta and Zr, Nb, and Hf were 0.643, 0.674, and 0.655, respectively. The mass fractions of the carbonate bound and residual state of Ta were 35% and 30%, respectively; previous studies concluded that Zr and Hf, and Nb and Ta are congeners in the periodic table with very similar electronic configurations and major geochemical parameters, and are often associated together in nature. It has been suggested that this group of metallic elements mainly occurs in inorganic minerals such as zircon or anatase in coal [43,44]. It is speculated that Zr, Nb, and Hf mainly exist in an inorganic bound state and occur in aluminosilicate minerals (clay minerals) kaolinite and illite. Ta exists in the inorganic bound state, and part of it exists in carbonate minerals calcite and dolomite.

The Be, Rb, and Cs had a high positive correlation coefficient with ash yield (r_Ad_ > 0.7, Table 5). The results of the sequential chemical extraction experiment showed that these elements were mainly in the residual and carbonate bound state, and the total mass fractions of the residual state and carbonate bound state were 89%, 94%, and 92%, respectively. Previous studies found that Be is mainly related to carbonate minerals containing Ca and Mn, followed by clay minerals [43]. Rb and Cs correlate well with K_2_O and Na_2_O (Table 5), indicating that Rb and Cs were probably trapped by K- and Na-bearing minerals (kaolinite and illite) or carbonates (calcite) [45].

Studies have shown that Li is mainly related to aluminosilicate; Li in coal mainly exists in clay minerals, and mica and tourmaline in the form of isomorphism [46,47]. The sequential chemical extraction experiment showed that Li mainly exists in the form of a residue (87%). It is speculated that Li mainly exists in the inorganic bound state in the coal seams of the Shanxi Formation. Sr mainly exists in the form of the carbonate bound state (39%), followed by an iron manganese oxidation state (29%), as well as a residual state (26%). Sr and Ca belong to alkaline earth metals with similar chemical properties. Therefore, Sr can replace Ca in the lattice of calcite and aragonite in the form of isomorphism [48]. At present, a variety of strontium-bearing minerals have been found in coal, including sulfate minerals (such as celestite), phosphate minerals (such as apatite, strontium, and phosphate), and carbonate minerals (such as strontite) [49]. It is speculated that Sr may partly exist in carbonate minerals in an inorganic bound state.

#### 4.3.2. Rare-Dispersed Elements

Ga, Ge, Cd, and Tl had a high positive correlation coefficient with ash yield (r_Ad_ > 0.68 Table 5). The results of the sequential chemical extraction experiment showed that Ga and Cd were mainly in a residual and carbonate bound state, and the total mass fractions of the residual state and carbonate bound state were 88% and 82%, respectively. The correlation coefficients of Ga (r = 0.472) and Cd (r = 0.614) with Al_2_O_3_ were positive. Ga in coal is mainly binds with inorganic minerals and has strong binding forces with clay minerals such as kaolinite and hydroxides such as boehmite. Moreover, Ga can replace Al in minerals with isomorphisms, and a small part of Ga can be stored in sulfide minerals [19]. It is speculated that Ga and Cd in the Shanxi formation mainly exist in the inorganic bound state, and may exist in carbonate minerals such as calcite, dolomite, and clay minerals kaolinite and illite. Tl correlated well with Na_2_O (r = 0.734) and K_2_O (r = 0.864), and had a positive correlation with Al_2_O_3_ (r = 0.455) and Fe_2_O_3_ (0.453). The sequential chemical extraction experiment results showed that the mass fraction of the Tl residue state was 46%, followed by the exchangeable state (27%), and the iron manganese oxidation state (15%), indicating that Tl mainly existed in an inorganic bound state, and some of it existed in clay minerals or pyrite. Few studies have been conducted on the occurrence status of Tl in coal, and the available data indicate that Tl in coal is mainly coeval with pyrite [33]. The migration distribution during washing is mainly controlled by clay minerals and pyrite [50]. Ge is mainly bound to organic matter in coal, and a small amount of Ge exists in an adsorbed state, including organic matter adsorption and clay mineral adsorption. A small amount of Ge may also be detected in sulfide and silicate minerals [3,49]. The results of sequential chemical extraction showed that the mass fraction of the organic bound state and residual state of Ge was 56% and 22%, respectively. Ge correlated well with Na_2_O (r = 0.611) and K_2_O (r = 0.627). It is presumed that Ge in the Shanxi Formation coal existed mainly in a combined form with organic matter and was partially adsorbed by clay minerals.

The occurrence state of Se in coal is very complex, sometimes dominated by an organic bound state, which may replace the sulfur in organic form. The Se of inorganic components in coal mainly exists in pyrite and sulfide, or enters the crystal lattice of aluminosilicate minerals, and may also be subordinate to tiny lead selenite compounds [49,51,52]. The correlation coefficient between Se and S was 0.471, and the correlation coefficient between Se and CaO was 0.528. The sequential chemical extraction experiment showed that Se mainly existed in the residual state (46%) and organic bound state (51%). It is speculated that Se in the Shanxi Formation coal not only existed in inorganic minerals (carbonate minerals, sulfide minerals, and pyrite) but was also closely related to organic minerals.

#### 4.3.3. Rare-Earth Elements

REYs in coal are mainly related to inorganic minerals such as clay minerals, phosphate, aluminophosphate, and carbonate [21,22,36]. Some of them occur in coal in the form of organic complexes [31]. The results of the sequential chemical extraction experiment showed that the mass fraction of REYs in the residual state was 71%, followed by an organic bound state, while the proportion of the iron manganese oxide state, carbonate state, and exchangeable ion state was relatively small. REYs correlated well with Al_2_O_3_ (r = 0.495), P_2_O_5_ (r = 0.556), and Fe_2_O_3_ (r = 0.471), suggesting that the occurrence state of REYs in the Shanxi Formation coal has possible associations with clay minerals and phosphate.

### 4.4. REY Geochemistry and Depositional Environment in Coal

#### 4.4.1. REY Geochemistry

The data of rare-earth elements in the upper crust UCC proposed by Taylor and McLennan were used for standardization [53] (Table 6). Three enrichment types were identified based on the three-fold geochemical classification of REYs [31]: L-type (light REY, La_N_/Lu_N_ > 1), M-type (medium REY, La_N_/Sm_N_ < 1, Gd_N_/Lu_N_ > 1), and H-type (heavy REY, La_N_/Lu_N_ < 1). In the Shanxi Formation, the (La/Lu)_N_ of 1–1, 1–2, 1–3, 1–5, 1–9, 1–10, and 3–10 was less than 1, which was rich in heavy rare-earth (H-type REY); the (La/Sm)_N_ of 1–9 and 1–10 was less than 1 and (Gd/Lu)_N_>1, which was rich in medium rare-earth (M–type REY); the remainder were rich in light rare-earth. The average value of (La/Lu)_N_ of the Shanxi Formation coal was 1.71 (range: 0.72–3.90), the average value of (La/Sm)_N_ was 1.72 (range: 0.87–2.84), and the average value of (Gd/Lu)_N_ was 1.72 (range: 0.46–3.28). In general, the distribution pattern of REYs in the Shanxi Formation was of the light REY enrichment type.

The abnormal values of Ce and Eu in coal samples were calculated by the following formula [54]:

$$\delta_{Ce}=\frac{Ce_{N}}{Ce_{N}{}^{*}}=\frac{Ce_{N}}{\sqrt{La_{N}\times Pr_{N}}}$$

$$\delta_{Eu}=\frac{E_{uN}}{Eu_{N}{}^{*}}=\frac{Eu_{N}}{\sqrt{Sm_{N}\times Gd_{N}}}$$

The average value of δCe from the Shanxi Formation coal in the Huainan coalfield was 0.96 (range: 0.68–1.43). In a sedimentary environment affected by seawater, Ce^3+^ is oxidized to Ce^4+^, which is an important factor rendering the coal to be poor in Ce. Therefore, the negative anomaly of Ce is usually regarded as one of the indicators of a marine sedimentary environment [55,56]. The negative or very weak negative Ce anomaly of coal preserved in carbonate rock series may be due to the joint influence of seawater and the basaltic sediment source area [57]. The Huainan Shanxi Formation coal exhibited a δCe < 1, showing a weak negative anomaly. Eu is the only rare-earth element that is sensitive to redox except for Ce. Eu^3+^ can be reduced to Eu^2+^ in a reducing environment, which leads to an obvious Eu anomaly relative to other REYs. The study showed that Eu originated from rocks in the sediment source area, and the coal samples affected by the terrigenous rocks had the characteristics of an Eu negative anomaly. Meanwhile, the interference of Ba to Eu should be considered during the test [57]. The average value of Ba/Eu in this test was 433.06 < 1000 (range: 130.33–993.34); thus, it can be considered that Ba had no interference with Eu. The average value of δEu in the Shanxi Formation coal was 0.73 (range: 0.52–1.16), indicating that the rare-earth elements in the Shanxi Formation coal were greatly affected by land-based control. The negative anomalies of Ce and Eu indicate that the REE content in the coal of the Huainan Shanxi formation was affected by seawater, but the influence was small, and the main source was the input of terrigenous clasts. This proves that the coal of Shanxi Formation in the Huainan coalfield was developed in the delta coal forming environment and gradually formed in the sedimentary environment from the delta front to the delta. The Y_N_/Ho_N_ ratio represents the geochemical decoupling. Y_N_/Ho_N_ < 1, Y was a negative anomaly; Y_N_/Ho_N_ > 1, Y was a positive anomaly; coal Y affected by seawater usually showed a positive anomaly [9]. The average value of Y_N_/Ho_N_ of the Shanxi coal in the deep part of Huainan was 1.11, which indicates that the sedimentary environment of the Shanxi Formation coal was affected by seawater.

#### 4.4.2. B and *w*(Sr)/*w*(Ba) as Indicators of Depositional Environment

Previous studies concluded that the Shanxi Formation of the Huainan Coalfield, as an early Permian coal-bearing formation in North China, was in an important period of marine-to-terrestrial transition, and that the upper part of the coal seam was deposited mainly in the delta plain environment dominated by fluvial action, while the lower part of the coal seam was mainly developed in the underwater delta and coastal sedimentary environment [24]. Previous sedimentological evidence of boreholes showed that coal seam 3 was sandwiched between black mudstones rich in coral fossils and white mica fragments, and was affected by a marine environment during the development of the coal seam [38].

**Figure 7:**
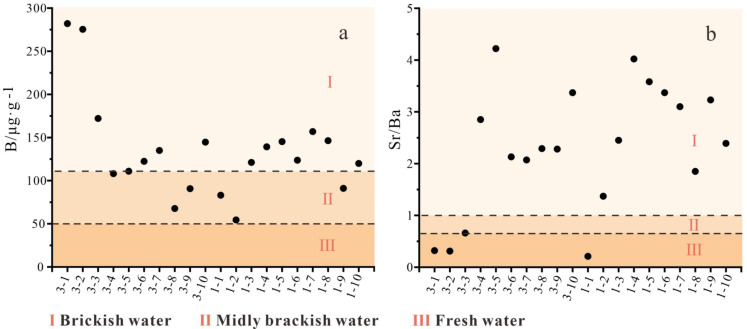
Variations in boron abundance (**a**) and the Sr/Ba ratio (**b**) of the Shanxi Formation in Huainan.

B is sensitive to changes in salinity. It is often used as a paleosalinity indicator of the coal-forming environments due to the good linear relationship between boron mass fraction and paleo-salinity [58,59,60]. In this study, 50 μg/g and 110 μg/g were classified into freshwater/mildly brackish water environments and mildly brackish water/marine water environments [61]. The B content in the Shanxi Formation coal in the Huainan coalfield was 54.4–282 μg/g, with an average content of 134.46 μg/g (Figure 7a). The average B content of the Shanxi formation coal were all within the range of mildly brackish-marine water environments proposed by Goodarzi and Swaine [62]. The B content showed a wide range in the coal seams of the Shanxi formation, indicating that the depositional environments were brackish but changed frequently. The coal development of the Shanxi Formation has been affected by seawater. The geochemical behaviors of Sr and Ba were different in the sedimentary environments. When influenced by seawater, the salinity increases. Due to the greater solubility of SeSO_4_ than that of BaSO_4_, the mobility of Sr is stronger than that of Ba in natural water; thus, Sr tends to enrich relatively to Ba in water. With increasing water salinity, Sr will precipitate in the form of sulfate as well. Therefore, the *w*(Sr)/*w*(Ba) in sediments is positively correlated with salinity and can be used as an indicator of paleosalinity. Previous studies have shown that *w*(Sr)/*w*(Ba) > 1 suggested brackish water deposition, *w*(Sr)/*w*(Ba) between 0.6 and 1 indicated mildly brackish water deposition, and *w*(Sr)/*w*(Ba) < 0.6 suggested terrestrial freshwater deposition [22,63]. The *w*(Sr)/*w*(Ba) of the Shanxi Formation ranged from 0.21 and 4.22 (avg. 2.31), indicating marine and marine-continental transitional environments (Figure 7b). The peak value of Sr/Ba ratio occurred for 3-5, 1-4, indicating that the deposition of coal seams was greatly influenced by seawater. The B content and Sr/Ba ratio confirm that the Shanxi Formation coal developed in the transitional phase semi-saline sedimentation of the deltaic sedimentary environment.

## 5. Conclusions

1. The Shanxi Formation coal in the Huainan coalfield was found to be characterized by ultra-low moisture, low ash, medium–high volatility, low-sulfur content, and high calorific value. The coal in the study area mainly consisted of clay minerals kaolinite and illite; oxide mineral quartz; carbonate minerals calcite and dolomite; a small amount of pyrite; sulfate minerals gypsum and jarosite.
2. Compared to the averages of Chinese and world hard coals, in the Shanxi Formation coal, rare-metal element Li and rare-dispersed element Se were enriched, and Ga and Ta were slightly enriched. Thus, the Shanxi formation coal is characterized by enriched concentrations of Li(Ta)−Se(Ga) assemblages. In terms of spatial distribution, the content of TREs in the roof, floor, and parting was higher than that in the coal seam. Affected by the sedimentary microenvironment, Li, Ta, and Se fluctuated greatly in the vertical direction of the coal seam. The results of the sequential chemical extraction experiment showed that the TREs in coal were closely related to inorganic matter. The reason for the relative enrichment of Se was related to the high proportion of its organic forms. The REY enrichment type was identified as an L-type because of the higher fractionation of light REYs than that of heavy REYs. Influenced by the depositional environment, the content of REYs in the Shanxi Formation coal increased gradually from coal seam 1 to coal seam 3. The results of the sequential chemical extraction experiment suggest that the REY in the Shanxi Formation coals was possibly associated with clay minerals in coal in an inorganic state, and may also be associated with phosphate and calcite in coal.
3. The C-value, B content, and Sr/Ba ratio in coals indicated that the Shanxi Formation coals in the Huainan coalfield were developed in the transitional-phase semi-saline sedimentation of the deltaic sedimentary environment. Furthermore, the results of the REY parameters suggest that the Shanxi Formation coal was affected by seawater, and REYs in coal were greatly supplied by terrigenous clastics. This evidence indicates that the Shanxi Formation coal in the Huainan coalfield was developed in the inter-deltaic coastal zone and lower deltaic plain environment. The complex sedimentary environment is an important reason for the varying occurrence states of TREs in the Shanxi Formation coal.

## Figures and Tables

**Table 1:** Sequential chemical extraction procedure used for TREs speciation.

Step	Speciation	Extractant	Extraction Conditions	Cellulose Filter
T1	Exchangeable	0.50 g sample + 10 mL NaOAc (1 M, pH = 8.2)	oscillate at room temperature of (25 ± 2) °C for 2 h, centrifuge	0.2 µm
T2	Carbonate	residue in T1 + HOAc(1 M, pH = 5.0)	stir until the reaction is complete at room temperature, then centrifuge	0.2 µm
T3	Fe-Mn oxides	residue in T2 + 20 mL of 0.3 M Na_2_S_2_O_4_ + 0.175 M Na-citrate + 0.025 H-citrate	occasional stirring at 96 ± 3 ^◦^C, then centrifuge	0.2 µm
T4	Organic	residue in T3 + ① 3 mLof 0.02 M HNO_3_ + 5 mL of H_2_O_2_ (pH = 2); ② 3 mL of 30% H_2_O_2_ (pH = 2, with HNO_3_); ③ 5 mL of 3.2 M NH_4_OAc	① 2 h at 85 ± 2 °C; ② 3 h at 85 ± 2 °C; ③ 0.5 h continuous stirring, then centrifuge.	0.2 µm
T5	Residual	residue recovered in T4 + 5 mLHNO_3_ + 5 mL HF	digestion at 110 °C to clear liquid, and then the cover was lifted at 90 °C to remove the acid	0.2 µm

**Table 2:** Coal quality analysis results of the Shanxi Formation coal in deep Huainan (%).

Sample No.	M_ad_	A_d_	V_daf_	Q (MJ/kg)	S_t,d_	S_p,d_	S_s,d_	S_o,d_
3-1	1.54	17.96	28.65	22.91	0.54	0.21	0.05	0.28
3-2	1.31	8.45	32.44	32.21	0.49	0.22	0.04	0.23
3-3	1.69	8.42	34.34	26.92	0.62	0.27	0.07	0.28
3-4	1.24	11.44	31.89	30.95	0.51	0.22	0.02	0.27
3-5	1.25	13.42	38.44	26.08	0.32	0.16	0.03	0.13
3-6	1.20	3.45	35.76	34.44	0.47	0.19	0.03	0.25
3-7	1.43	6.80	31.93	27.78	0.59	0.24	0.05	0.30
3-8	1.25	4.00	33.95	34.18	0.49	0.23	0.04	0.22
3-9	1.24	6.13	36.19	33.25	0.45	0.20	0.03	0.22
3-10	1.57	7.77	33.95	26.99	0.43	0.17	0.01	0.25
1-1	2.05	29.04	31.36	19.10	0.40	0.19	0.01	0.20
1-2	1.96	8.06	29.51	27.10	0.42	0.16	0.02	0.24
1-3	2.00	13.23	30.59	26.80	0.36	0.15	0.05	0.16
1-4	2.37	12.93	31.35	27.07	0.52	0.25	0.04	0.23
1-5	1.80	10.60	32.34	26.12	0.46	0.21	0.03	0.22
1-6	2.06	5.08	30.44	28.43	0.52	0.22	0.02	0.28
1-7	2.01	8.84	31.83	27.05	0.56	0.24	0.06	0.26
1-8	2.04	5.68	33.17	27.15	0.64	0.26	0.04	0.34
1-9	2.74	14.72	33.54	25.21	0.54	0.27	0.04	0.23
1-10	2.93	21.75	33.43	22.13	0.71	0.41	0.03	0.27
Average	1.78	10.89	32.76	27.59	0.50	0.22	0.04	0.24
Max	2.93	29.04	38.44	19.10	0.71	0.41	0.07	0.34
Min	1.20	3.45	28.65	34.44	0.32	0.15	0.01	0.13

M_ad_, moisture; A_d_, ash yield; V_daf_, volatile matter yield; S_t_, total sulfur; S_p_, pyritic sulfur; S_s_, sulfate sulfur; S_o_, organic sulfur.

**Table 3:** Content range of major oxides in the coal of the Shanxi Formation in Huainan (%).

Sample	Project	Na_2_O	MgO	Al_2_O_3_	SiO_2_	K_2_O	CaO	Fe_2_O_3_	P_2_O_5_	TiO_2_	C
	Min	0.02	0.09	2.28	4.01	0.01	0.30	0.31	0.01	0.05	0.14
Shanxi Formation	Max	0.28	0.86	7.77	7.86	1.92	1.60	1.99	0.03	0.77	0.35
	Ave	0.07	0.46	4.93	5.43	0.25	0.83	1.24	0.02	0.27	0.25
China	Ave	0.16	0.22	5.98	8.47	0.19	1.23	4.85	0.10	0.33	

C = [w(CaO) + w(MgO) + w(Fe_2_O_3_)]/[w(SiO_2_) + w(Al_2_O_3_)].

**Table 4:** Test results of TREs in coal of the Shanxi Formation in Huainan (μg/g).

Elements	This Study			Northern China		Chinese Coal		World Hard Coal
	Min	Max	AM	AM	GM	AM	Sample No.	AM
Li	11.58	119	67.56	43.91	33.5	31.8	1274	12
Be	0.13	23.42	1.97	2.05	1.69	2.13	1198	1.6
Rb	0.52	57.41	6.61	1.59	0.98	9.24	1114	14
Sr	29.83	899.6	193.4	192.99	116.51	140.2	2075	110
Zr	10.5	150.9	41.14	188.28	150.9	89.3	1238	36
Nb	0.34	16.54	4.27	6.87	5.48	9.47	974	3.7
Cs	0.1	1.83	0.26	0.39	0.11	1.13	1208	1
Hf	0.23	4.95	1.27	5.07	4.11	3.82	1320	1.2
Ta	0.62	0.99	0.79	0.6	0.4	0.66	1336	0.28
Ga	4.64	35.76	13.67	12.57	11.17	6.52	1986	5.8
Ge	1.17	7.26	2.71	nd	nd	2.96	3189	2.2
Se	2.06	12.58	6.92	2.01	0.97	2.47	1537	1.3
Cd	0.06	1.18	0.32	0.11	0.08	0.24	1303	0.22
Tl	0.02	0.67	0.13	0.22	0.13	0.48	1018	0.63
B	54.4	282	134.46	nd	nd	53	1048	52
Ba	33.82	301.1	90.48	121.59	57.92	159	1205	150
REY	31.06	80.57	51.34	nd	nd	136	nd	68.27

Min: minimum; Max: maximum; AM: arithmetic means; GM: geometric mean; nd: no data.

**Table 5:** Pearson correlation coefficient of single element and ash yield and selected elements or element combinations in coal seams of the Shanxi Formation.

Correlation Coefficients with Ash Yield				
r_Ad–Be_ = 0.746 **	r_Ad–Rb_ = 0.711 **	r_Ad–Zr_ = 0.726 **	r_Ad–Nb_ = 0.623 **	r_Ad–Cd_ = 0.760 **
r_Ad–Cs_ = 0.746 **	r_Ad–Hf_ = 0.710 **	r_Ad–Ga_ = 0.683 **	r_Ad–Tl_ = 0.841 **	r_Ad–Ge_ = 0.707 **
Correlation coefficient with Al_2_O_3_				
r_Al2O3–Be_ = 0.451 *	r_Al203–Zr_= 0.613 **	r_Al2O3–Nb_ = 0.596 **	r_Al2O3–Hf_ = 0.562 **	r_Al2O3–Tl_ = 0.455 *
r_Al2O3–Ga_ = 0.472 *	r_Al2O3–Cd_ = 0.614 **			
Correlation coefficient with K_2_O				
r_K2O–Be_ = 0.873 **	r_K2O–Cs_= 0.940 **	r_K2O–Ge_ = 0.627 **	r_K2O–Tl_ = 0.864 **	r_K2O–Rb_ = 0.870 **
Correlation coefficient with Na_2_O				
r_Na2O–Be_ = 0.887 **	r_Na2O–Cs_= 0.865 **	r_Na2O–Ge_ = 0.611 **	r_Na2O–Tl_ = 0.734 **	r_Na2O–Rb_ = 0.771 **
Correlation coefficients with selected element combinations				
r_Ta–Zr_ = 0.643 **	r_Ta–Nb_ = 0.674 **	r_Ta–Hf_ = 0.655 **	r_Se–CaO_ = 0.528 *	r_Se–s_= 0.471 *
r_REY–P2O5_ = 0.556 **	r_REY–CaO_ = 0.553 *	r_REY–S_ = 0.471 *	r_REY–Al_ = 0.495 *	r_Tl–Fe2O3_ = 0.453 *

* Correlation is significant at the 0.05 level (2-tailed). ** Correlation is significant at the 0.01 level (2-tailed).

**Table 6:** Geochemical parameters of rare-earth elements in the Shanxi Formation coal seam from the Huainan coalfield.

Sample No.	LREY	MREY	HREY	∑REY	L/M	M/H	L/H	δCe	δEu	(La/Lu)_N_	(La/Sm)_N_	(Gd/Lu)_N_	(Y/Ho)_N_
3-T	88.72	12.51	2.17	103.41	7.09	5.75	40.80	0.87	0.98	1.89	1.65	2.21	0.74
3-1	63.43	11.22	3.09	77.74	5.65	3.63	20.52	0.68	0.61	1.32	1.51	0.46	1.06
3-2	38.16	4.38	0.54	43.08	8.71	8.11	70.60	0.89	0.63	3.40	2.17	2.73	1.31
3-3	46.72	7.82	1.44	55.97	5.98	5.44	32.51	0.84	0.79	1.95	2.13	1.67	1.13
3-4	69.73	7.73	1.09	78.54	9.03	7.10	64.07	0.84	0.82	3.90	2.60	2.57	1.16
3-5	46.35	6.24	1.02	53.62	7.42	6.10	45.26	0.68	0.75	3.45	2.84	2.02	0.89
3-6	34.11	5.78	0.99	40.87	5.90	5.87	34.63	1.26	0.84	1.92	2.18	2.01	0.85
3-7	46.02	6.51	1.20	53.73	7.07	5.45	38.49	0.72	0.59	2.47	2.21	1.53	1.22
3-8	52.03	5.19	1.05	58.27	10.02	4.94	49.54	1.03	0.62	2.62	2.62	1.30	1.13
3-9	33.30	6.70	0.67	40.67	4.97	9.99	49.63	1.03	1.16	1.69	2.26	3.28	1.45
3-10	35.19	11.36	1.51	48.06	3.10	7.51	23.28	1.07	0.74	0.95	1.32	2.84	1.29
3-B	71.65	15.53	2.89	90.07	4.61	5.37	24.78	0.88	1.09	1.00	0.81	1.85	0.92
1-T	87.26	7.32	1.15	95.73	11.91	6.35	75.71	1.73	0.97	3.99	2.67	1.53	1.45
1-1	27.94	2.83	0.81	31.57	9.89	3.51	34.67	1.29	0.89	0.72	0.89	0.69	0.53
1-2	29.36	5.12	1.02	35.50	5.74	5.00	28.71	0.86	0.67	0.95	1.28	1.28	0.94
1-P	19.43	8.65	2.50	30.58	2.25	3.46	7.78	0.76	2.10	0.16	0.73	0.28	0.59
1-3	37.19	9.07	1.77	48.02	4.10	5.13	21.06	0.81	0.73	0.90	1.08	1.33	1.06
1-4	46.67	10.62	1.93	59.22	4.39	5.50	24.15	0.72	0.87	1.47	1.72	1.41	1.19
1-5	32.32	6.84	1.29	40.45	4.73	5.31	25.10	0.93	0.78	0.97	1.09	1.51	1.11
1-6	39.72	6.95	1.28	47.95	5.71	5.43	31.00	0.86	0.74	1.51	1.77	1.52	1.16
1-7	37.90	5.91	1.10	44.91	6.42	5.36	34.37	1.43	0.61	1.33	1.52	1.67	1.14
1-8	25.19	5.02	0.83	31.04	5.02	6.03	30.27	1.22	0.66	1.19	1.28	1.57	1.31
1-9	48.16	12.77	2.37	63.30	3.77	5.40	20.35	1.03	0.61	0.85	0.87	1.58	1.06
1-10	53.33	16.08	2.86	72.27	3.32	5.63	18.66	1.05	0.52	0.73	0.93	1.52	1.18
1-B	85.74	7.63	1.22	94.59	11.24	6.28	70.51	1.74	0.71	3.35	1.42	2.00	1.18
Average	42.14	7.71	1.39	51.24	6.05	5.82	34.84	0.96	0.73	1.71	1.72	1.72	1.11

N, REY are normalized by upper continental crust (UCC).

## Data Availability

The data collected are property of our research center but will be made available by the corresponding author when requested.
